# Development of an Advanced Multi-Layer Digital Twin Conceptual Framework for Underground Mining

**DOI:** 10.3390/s25216650

**Published:** 2025-10-30

**Authors:** Carlos Cacciuttolo, Edison Atencio, Seyedmilad Komarizadehasl, Jose Antonio Lozano-Galant

**Affiliations:** 1 Department of Civil Engineering, Universidad de Castilla-La Mancha, Av. Camilo Jose Cela s/n, 13071 Ciudad Real, Spain; joseantonio.lozano@uclm.es; 2 School of Civil Engineering, Pontificia Universidad Católica de Valparaíso, Av. Brasil 2147, Valparaíso 2340000, Chile; edison.atencio@pucv.cl; 3 Department of Civil and Environmental Engineering, Universitat Politècnica de Catalunya, BarcelonaTech, C/Jordi Girona 1-3, 08034 Barcelona, Spain

**Keywords:** digital twins, underground mine, monitoring, prediction, simulation, lifecycle management, decision support system, expert panel survey

## Abstract

Digital mining has been evolving in recent years under the Industry 4.0 paradigm. In this sense, technological tools such as sensors aid the management and operation of mining projects, reducing the risk of accidents, increasing productivity, and promoting business sustainability. DT (Digital Twin) is a technological tool that enables the integration of various Industry 4.0 technologies to create a virtual model of a real, physical entity, allowing for the study and analysis of the model’s behavior through real-time data collection. A digital twin of an underground mine is a real-time, virtual replica of an actual mine. It is like an extremely detailed “simulator” that uses data from sensors, machines, and personnel to accurately reflect what is happening in the mine at that very moment. Some of the functionalities of an underground mining DT include (i) accurate geometry of the real physical asset, (ii) real-time monitoring capability, (iii) anomaly prediction capability, (iv) scenario simulation, (v) lifecycle management to reduce costs, and (vi) a support system for smart and proactive decision-making. A digital twin of an underground mine offers transformative benefits, such as real-time operational optimization, improved safety through risk simulation, strategic planning with predictive scenarios, and cost reduction through predictive maintenance. However, its implementation faces significant challenges, including the high technical complexity of integrating diverse data, the high initial cost, organizational resistance to change, a shortage of skilled personnel, and the lack of a comprehensive, multi-layered conceptual framework for an underground mine digital twin. To overcome these barriers and gaps, this paper proposes a strategy that includes defining an advanced, multi-layered conceptual framework for the digital twin. Simultaneously, it advocates for fostering a culture of change through continuous training, establishing partnerships with specialized experts, and investing in robust sensor and connectivity infrastructure to ensure reliable, real-time data flow that feeds the digital twin. Finally, validation of the advanced multi-layered conceptual framework for digital twins of underground mines is carried out through a questionnaire administered to a panel of experts.

## 1. Introduction

In underground mining, there has always been a challenge: safe conditions where the risk of accidents is permanent. In this sense, a brief introduction is presented below to understand the research problem and thus visualize alternative solutions for digital twins with the implementation of sensors and the Internet of Things (IoT) that could improve some aspects of complex working environmental conditions.

### 1.1. Operational Challenges in Underground Mines

Underground mining faces significant challenges related to safety, operational efficiency, and environmental sustainability. These aspects are critical to ensuring viable operations in an environment characterized by extreme conditions and technical limitations. These challenges are examined below in the context of risk reduction, increased productivity, and sustainable development [1].

**Figure 1:**
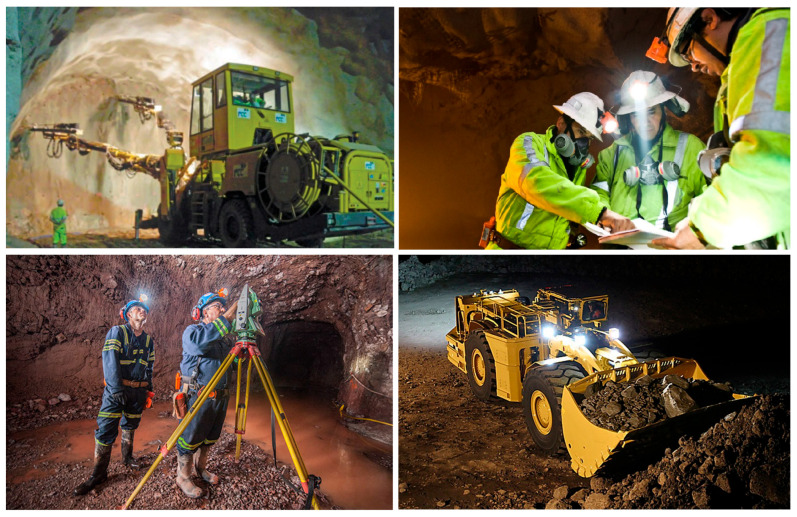
Typical environmental conditions in an underground mine.

First, operational risk reduction should be mentioned, as underground mining is one of the riskiest industrial activities due to a number of hazards, including cave-ins, gas explosions, and exposure to hazardous materials, among others [2] (see Figure 1). Technological advances have made it possible to address these risks through the following: (i) Real-time geotechnical monitoring: the implementation of sensors to measure structural deformations and vibrations has significantly reduced accidents in deep mines. (ii) Automation and remote operation: the remote operation of equipment, such as drills and loaders, reduces worker exposure to high-risk areas. (iii) AI-based predictive systems: analytical models that anticipate critical events, such as cave-ins or toxic gas leaks, have proven effective in preventing accidents [3].

Secondly, it is important to discuss increased productivity, where productivity in underground mining is influenced by factors such as process efficiency, equipment availability, and optimization of material transportation. Some notable approaches to enhance or improve productivity include the following: (i) Digital twin integration: these systems allow mining operations to be simulated and extraction processes optimized, reducing associated times and costs. (ii) Use of autonomous machinery: automated vehicles and loading and unloading systems optimize operations by reducing interruptions and increasing equipment utilization. (iii) Smart mining: real-time data analysis to dynamically adjust operations has increased efficiency in several underground operations [4].

Third and finally, the development of sustainability should be mentioned, where underground mining has a significant impact on the environment and local communities. Achieving sustainable operations requires adopting responsible practices in resource use and waste management. Key approaches include the following: (i) Emissions reduction: the electrification of equipment and the use of renewable energy sources have contributed to reducing the carbon footprint of underground operations. (ii) Water management: advanced water recycling and monitoring systems to prevent contamination of nearby aquifers have improved environmental practices. (iii) Environmental restoration: initiatives to remediate affected areas and comply with international sustainability standards have strengthened the social license to operate [5].

Despite progress, challenges such as a lack of interoperability between technologies, high initial implementation costs, and resistance to organizational change persist. However, opportunities for improvement through technologies such as the Internet of Things (IoT), artificial intelligence (AI), and digital twins (DTs) are significant. These tools not only optimize processes but also improve safety and reduce environmental impact [6].

### 1.2. Why Are Underground Mine Digital Twins Needed? A Growing Simulation Issue

Digital twins are essential in underground mining due to their ability to address critical challenges such as safety, operational optimization, and sustainability. These technological tools offer accurate virtual representations of physical systems that allow for real-time monitoring, simulation, and optimization of mining operations [7]. The main reasons for their importance in this sector are discussed below.

Underground mining faces inherent risks such as cave-ins, toxic gas accumulation, and exposure to unstable geotechnical conditions. Digital twins help mitigate these risks through the following [8,9]:

- Real-time monitoring: integrating sensors with digital twins provides constant data on drift stability, hazardous gas levels, and other critical factors.
- Predictive simulations: virtual models can predict adverse events such as structural failures, enabling preventive measures to be implemented before accidents occur.
- Reducing human exposure: by operating equipment remotely through the digital twin, workers can avoid hazardous areas, significantly improving workplace safety.

**Figure 2:**
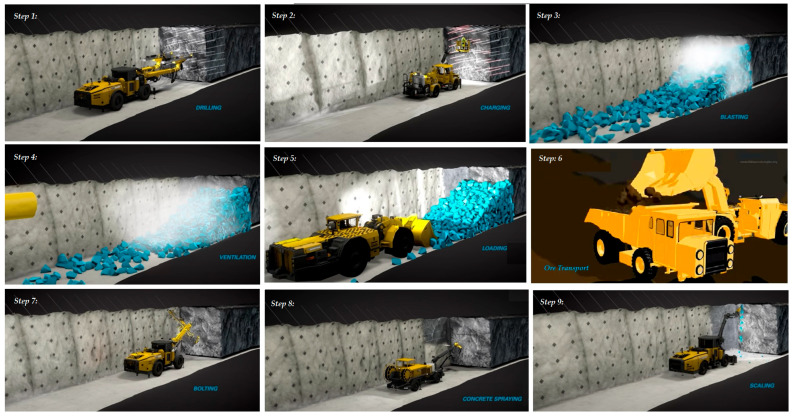
Main activities developed in a typical underground mine. **Step 1**: drilling; **Step 2**: charging; **Step 3**: blasting; **Step 4**: ventilation; **Step 5**: loading; **Step 6**: ore hauling; **Step 7**: bolting; **Step 8**: concrete spraying; and **Step 9**: scaling.

The complexity of underground mining operations demands efficient management of resources and processes. Digital twins are essential for the following [10,11]:

- Efficient equipment management: they enable real-time monitoring of equipment status, facilitating predictive maintenance and reducing downtime (see Figure 2).
- Simulation of operating processes: with digital twins, it is possible to simulate mining designs and machinery movements to optimize workflow and minimize disruptions (see Figure 2).
- Data-driven decision-making: by consolidating and analyzing operational information in real time, digital twins enable operators to make dynamic adjustments that increase productivity (see Figure 2).

Underground mining faces increasing pressure to reduce its environmental impact and meet sustainability standards. Digital twins contribute to this goal in the following ways [12,13]:

- Reducing energy consumption: by optimizing operations and reducing downtime, digital twins reduce energy consumption, indirectly reducing carbon emissions.
- Efficient water management: simulations of water flow and drainage make it possible to prevent contamination of nearby aquifers and optimize the use of this critical resource.
- Environmental restoration: virtual models help plan land reclamation strategies, aligning with responsible mining principles.

Digital twins not only address immediate problems but are also a key component in the digital transformation of underground mining withing the Industry 4.0 context. They integrate advanced technologies such as the IoT, AI, and data analytics, creating an interconnected technological ecosystem. This integration enables the following [14,15]:

- Process standardization: improves interoperability between existing systems and technologies.
- Advanced predictive capabilities: the combination of AI and digital twins allows for the prediction of long-term trends in operations, improving strategic planning.
- Advanced visualization: augmented reality (AR) and virtual reality (VR) tools integrated with digital twins allow operators to interact with detailed mine models to assess risks and plan operations.

### 1.3. Why Are Underground Mine Digital Twins and Automation More Cost-Effective and Safer?

Digital twins and automation make underground mining more profitable by optimizing operations, reducing labor and maintenance costs, and enabling data-driven decision-making. At the same time, they significantly improve safety by minimizing human exposure to hazards, enabling real-time monitoring, and optimizing emergency response capabilities. The profitability of the mining process is also improved, as operations are optimized and real-time monitoring and control are enabled, resulting in safer and more efficient mining processes [16].

These advantages make them especially valuable in the challenging underground mining environment. One of these challenges is the cost-effectiveness of the mine, where three aspects stand out: (i) Operational efficiency: Digital twins enable the simulation, prediction, and optimization of mining processes, reducing downtime and improving resource allocation. Automation minimizes manual intervention, streamlining workflows and reducing labor costs. (ii) Maintenance and reliability: automated systems and digital twins can predict equipment failures and optimize maintenance schedules, reducing unexpected breakdowns and costly repairs. (iii) Data-driven decisions: real-time data from IoT sensors and devices feeds digital twins, enabling better decision-making and process optimization, resulting in lower operating costs [17]. Another very important advantage of DT refers to safety improvements, where three key aspects can be highlighted: (i) Remote operations: automation and digital twins enable remote control and monitoring, reducing worker exposure to dangerous underground conditions. (ii) Real-time hazard detection: sensors and digital twins provide continuous monitoring of hazards such as gas leaks, equipment failures, or structural instabilities, enabling rapid response. (iii) Emergency management: digital twins can simulate emergency scenarios and support the design of safety zones and emergency shutdown systems, improving preparedness and response [18].

Finally, another key advantage is related to improved monitoring and integration, where two fundamental aspects can be highlighted: (i) comprehensive surveillance—the integration of IoT, sensors, and digital twins provides real-time visibility of the entire mining operation, supporting both safety and efficiency—and (ii) interoperability—digitalization enables seamless communication between different systems and devices, improving coordination and reducing errors [19].

### 1.4. Scope of the Review

Despite the benefits of DTs in underground mines, no conceptual framework has been presented in the literature. To fill this gap, this paper aims to develop a comprehensive review of DTs applied to underground mining from the perspective of mining project management and operation. It provides an overview of state-of-the-art DT components and classifies them according to their purposes, functionalities, and integrated technologies for underground mining. Subsequently, based on the research results and identified gaps, this article proposes an advanced multi-layered DT conceptual framework with an optimized management plan supported by decision support systems, which could overcome the inherent vulnerability of human nature in the planning process due to the different levels of experience and knowledge of the decision-makers involved.

As background to this paper, two previous research studies were conducted: the first on the application of low-cost sensors in mining [20] and the second on the LoRaWAN IoT connectivity technology applied to underground mining [5]. These two studies form the basis for this paper and allow for the convergence of knowledge towards the development of digital twins for underground mining.

The main research questions (RQs) to be answered are presented below:

- RQ1: What does an underground mine digital twin mean?
- RQ2: What are the components of an underground mine digital twin?
- RQ3: What are the functionalities of an underground mine digital twin?
- RQ4: What is an advanced multi-layered digital twin conceptual framework for underground mining?
- RQ5: Is the advanced multi-layered digital twin conceptual framework valid for an expert panel?
- RQ6: What are the future directions for underground mining digital twins?

The structure of the paper is as follows: (i) introduction, (ii) literature review, (iii) definition of an underground mine digital twin and its components, (iv) advanced multilayer conceptual framework for underground mine digital twins, (v) functionalities of the advanced multilayer conceptual framework for underground mine digital twins, (vi) validation by an expert panel, (vii) discussion, and (viii) conclusions.

## 2. Literature Review of Digital Twins in Underground Mines

This section presents a literature review, first describing the implemented research methodology and then presenting the current state of the art on the investigated topic.

### 2.1. Research Methodology

**Figure 3:**
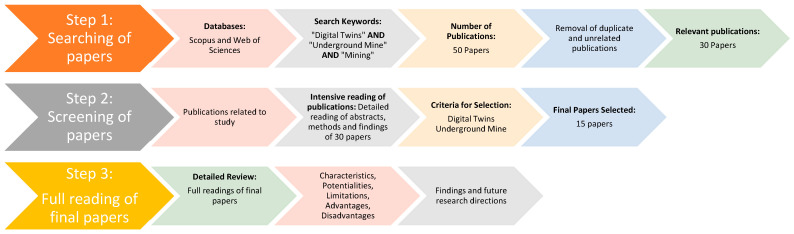
Methodological procedure for a literature review of digital twins in underground mine.

The literature review for this research was conducted using a three-phase methodology to accurately identify relevant publications. The process, depicted in Figure 3, consisted of three steps: (i) an initial search in Scopus and Web of Science, (ii) a subsequent selection phase, and (iii) a final content analysis.

#### 2.1.1. Preliminary Search

In the first stage, a literature search was performed for the period 2020 (March)–2025 (July) using the Scopus and Web of Science (WoS) databases. This selection was based on their status as leading, comprehensive global repositories that index high-quality journals featuring detailed case studies. The search strategy targeted the ‘Title-Abstract’ field in Scopus and the ‘Topic’ field in WoS. The use of other specialized databases was outside the scope of this research.

#### 2.1.2. Literature Screening

To guarantee the quality of the literature, the selection process was conducted in three distinct stages (see Figure 3). First, a search using the study’s keywords yielded approximately 50 publications. Next, this initial pool was refined by limiting publication types to “articles” and “review articles” and removing duplicates, resulting in a preliminary list of 30 documents.

To complement the selection process, explicit inclusion and exclusion criteria were implemented during the comprehensive evaluation of the 30 papers. The inclusion criteria required that publications (i) specifically address the application of emerging technologies (such as artificial intelligence, the Internet of Things, or automation) in underground mining operations; (ii) present empirical results, case studies, or validated methodological frameworks; and (iii) be published after 2015 to ensure technological relevance. Conversely, articles that only mentioned these technologies tangentially, studies focused exclusively on the exploration phase of mining without addressing operation, and publications not available in full text for in-depth analysis were excluded.

The final evaluation was conducted through a thorough critical reading of each publication, systematically applying the aforementioned criteria. This scrutiny made it possible to precisely identify the 15 publications that not only met all the requirements but also contributed substantial findings and conclusions directly related to the research objectives. Figure 3 illustrates this filtering process, showing how each stage refined the documentary corpus, while Table 1 details the database search parameters, providing complete transparency and reproducibility to the selection method, which significantly strengthens the validity of the literature review.

The data extraction index provides key information from papers considering technological advances and integration with the Industry 4.0 concept.

#### 2.1.3. Literature Study

In this stage, the ideas are synthesized and then the selected publications are thoroughly analyzed. First, existing techniques and experiences are summarized. Second, the basic architectural structure of digital twins for underground mines is proposed. Finally, the types of digital twins commonly used in underground mines are reviewed, the current state of development is analyzed, knowledge gaps are identified, and possible future research directions are proposed (see Figure 3).

### 2.2. State of the Art

This section presents a review of the state-of-the-art application of digital twins in industry, considering the mining industry. A summary of the identified papers is presented in Table 2 indicating the authors’ names, year of publication, publication title, a brief overview, and Industry 4.0 tools related to digital twins.

Among the 15 publications presented in the table above, it is possible to highlight four of them, directly linked to digital twins applied to the mining industry.

The article presented by Van Eyk and Heyns [21] teaches an innovative conceptual framework for the creation and understanding of digital twins in the mining industry. The authors address the existing confusion surrounding the concept and develop a structure that classifies and defines the dimensions, models, properties, and types of digital twins applicable to the mining sector. They highlight the usefulness of their framework in the planning and design of these systems, facilitating the identification of appropriate technologies and models for different use cases, such as underground mines and large-scale operations. Furthermore, they exemplify their approach by analyzing two case studies: a digital twin of a cutterhead and one of mining operation planning, demonstrating its versatility and applicability. Finally, the paper emphasizes the importance of standardization and shared knowledge to accelerate the implementation of digital twins in mining, providing tools that help reduce uncertainties and improve decision-making.

Furthermore, the article presented by Cranford [22] analyzes the potential application of digital twin technology in the mining industry to improve the measurement of and compliance with ESG (environmental, social, and governance) dimensions. Based on thirty case studies and theoretical applications in related industries, the research concludes that digital twins can be used in all phases of mining projects to enhance ESG objectives, offering more accurate, transparent, and consistent measurement. Furthermore, this work highlights that, although there are challenges related to interoperability, standardization, and data availability, investment in related technologies and advances in the field suggest that the time to implement these systems is coming. According to this work, successful implementation will require trained personnel and clear regulatory frameworks, but its use has the potential to transform ESG management and reporting, facilitating a more proactive and reliable approach to meeting sustainable goals in the mining sector.

The third work in the literature related to DTs was published by Nobahar et al. [23], highlighting their key benefits: increased profitability, improved performance, optimized safety, and environmental compliance. This work presents specific applications in mineral exploration, drilling, transportation, and processing that are reviewed, identifying critical data, tools, and methods for developing digital twins with simulation, prediction, and optimization capabilities. Despite the increasing use of technologies such as IoT, challenges such as geological variability, high costs, and dependence on human oversight persist. To address these issues, this paper suggests that greater integration of IoT sensors and devices can drive innovation and efficiency across the mining value chain. In conclusion, the study offers valuable insights for achieving more efficient and sustainable mining systems through the advanced adoption of AI and digital twins.

Finally, the article by Bazi et al. [24] addresses the impact of emerging technologies such as the IoT, Big Data, cloud computing, AI, and DTs on the mining industry. This work highlights how DTs have gained relevance due to their ability to improve efficiency, productivity, and sustainability through monitoring, simulating, and predicting failures and performance. Furthermore, it highlights the growing demand for customized products, which requires effective management of the entire product’s lifecycle, from design to end-of-life. According to this study, the main challenge is the successful integration of DTs into the mining industry. The article explores state-of-the-art DT case studies, focusing on their concept, design, and development. It also analyzes the reference architecture model for DTs in Industry 4.0 and proposes a mining-specific multi-layer architectural framework to guide future research and practical applications. In summary, the text provides an overview of the potential of DT technology in mining, its implementation challenges, and a theoretical framework for its future development.

## 3. Definition of Underground Mine Digital Twins and Components

The following section defines the concept of a digital twin for underground mining, presents the related technologies, and finally outlines the components of the digital twin.

### 3.1. Definition of Underground Mine Digital Twins and Related Technologies

#### 3.1.1. Digital Twins

In the context of underground mining, digital twins are defined as dynamic virtual representations of the physical systems, processes, and environments that comprise a mining operation. These digital replicas integrate real-time data from sensors and connected devices, allowing mining conditions and operations to be monitored, analyzed, and simulated with high accuracy [36].

A digital twin combines 3D and 4D models, real-time data analytics, and predictive simulations to provide a comprehensive view of underground processes. For example, these technologies are used to monitor equipment status, assess the geotechnical stability of drifts, and optimize ventilation systems. Furthermore, digital twins enable the integration of advanced tools such as AI and ML, facilitating informed, data-driven decision-making [37].

**Figure 4:**
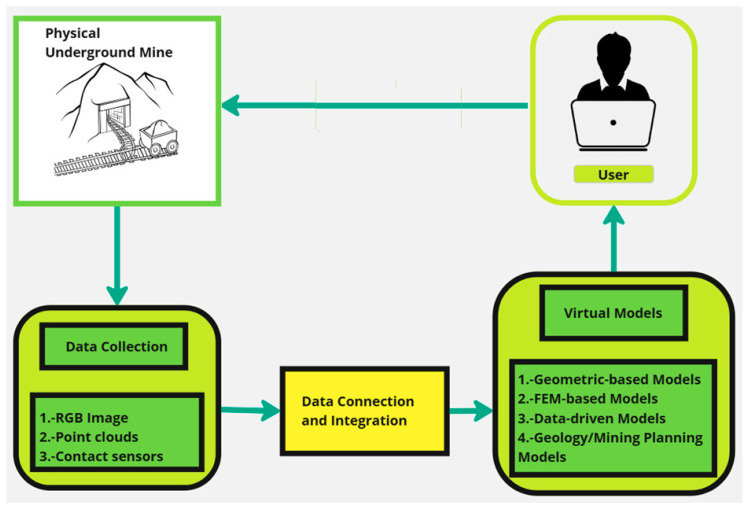
Elementary diagram of underground mine digital twin operation.

According to the literature, the ideal underground mine DT encompasses five main primary components, as shown in Figure 4, including the physical entity (underground mine), data collection, data connection/integration, virtual models, and the user as the final operator and manager of the underground mine.

According to Figure 4, the process begins in a physical underground mine, represented by a schematic drawing of a mine with a mine car on rails. Data is collected from the mine, represented by the “Data Collection” block, which includes RGB images, point clouds, and contact sensor data. This information is integrated through the “Data Connection and Integration” block and used to create virtual models. Virtual models are divided into four types: geometry-based models, finite element models (FEMs), data-driven models, and geological/mine planning models. These virtual models (shown in Figure 4) are also critical for reducing risks in complex environments, minimizing human exposure in hazardous areas, and optimizing resources such as water and energy. On the other hand, digital twins offer advanced simulation capabilities, allowing for testing of hypothetical scenarios to improve planning and operational performance [38,39]. Finally, a user interacts with the system via a laptop.

#### 3.1.2. Sensors

In underground mining, sensors are fundamental components for the development and operation of digital twins. These devices collect real-time data on the environment, operating conditions, and equipment performance, serving as a bridge between physical systems and their digital representations. Sensors enable digital twins to be dynamic and faithfully reflect the state of the mining operation, facilitating monitoring, decision-making, and strategic planning [20].

Some of the most common types of sensors in underground mining are the following: (i) Environmental sensors: these measure temperature, humidity, pressure, and concentrations of hazardous gases such as methane and carbon dioxide, ensuring safe conditions for workers. (ii) Geotechnical sensors: these record deformations, vibrations, and movements in underground structures, helping to prevent collapses or failures in mineshafts. (iii) Equipment sensors: these monitor parameters such as energy consumption, vibration, and wear on heavy machinery, enabling predictive maintenance strategies [5,20].

Furthermore, the data collected by sensors is integrated into digital twins to enable advanced functionalities, such as the following [40]:

- Real-time monitoring: sensors continuously send data to digital models, allowing the current state of operations to be assessed and anomalies to be detected immediately.
- Predictive simulations: historical and real-time data is used to predict failures, optimize processes, and simulate scenarios such as changes in ventilation or extraction configurations.
- Improved safety: geotechnical and environmental sensors provide early warnings in the event of risks, such as structural instabilities or dangerous gas levels.

The use of sensors is also combined with technologies such as IoT and cloud computing, improving connectivity and data analysis, even in remote or hard-to-reach mining environments [41].

#### 3.1.3. IoT

The IoT refers to the interconnection of devices and sensors through networks that enable the collection, exchange, and analysis of data in real time. These devices are equipped with communication and processing technologies that enable them to interact with each other and with control systems, facilitating the automation and optimization of processes in multiple industries [42].

In the context of underground mining, the IoT enables remote monitoring, real-time data transmission, and the integration of information from diverse sources, which is essential for creating accurate and dynamic digital twins.

Some of the most important IoT applications in digital twins for underground mining are the following [43]:

- Real-time data collection: IoT devices, such as environmental, geotechnical, and equipment sensors, collect critical information on variables such as temperature, gas concentration, tunnel stability, and machinery performance. This data is transmitted to the digital twin to reflect the status of underground operations.
- Remote monitoring and control: The IoT enables real-time connection between physical systems and their digital representations. This enables operators to monitor underground mine conditions from remote locations and make informed decisions to minimize risks and optimize processes.
- Predictive maintenance optimization: IoT sensors installed on machinery and equipment collect data on their operating status, such as vibrations, temperature, and wear. The digital twin uses this information to predict failures and schedule preventive maintenance, reducing downtime and operating costs.
- Integration with AI algorithms and simulations: Information collected by IoT devices is used in predictive simulations within the digital twin. For example, hypothetical scenarios, such as ventilation adjustments or changes in extraction methods, can be modeled to assess their impact on safety and productivity.
- Sustainability management: IoT devices help monitor the consumption of resources such as energy and water, as well as measure the environmental impact of operations. Digital twins use this information to design more sustainable and efficient strategies.

The IoT acts as a fundamental enabler for digital twins in underground mining, providing the data necessary for their creation and real-time operation. Furthermore, combining the IoT with technologies such as cloud computing and artificial intelligence expands the capabilities of analysis, simulation, and automation in complex mining environments [44].

#### 3.1.4. Artificial Intelligence

AI is a field of computer science that seeks to develop systems capable of performing tasks that normally require human intelligence, such as learning, decision-making, perception, and natural language processing. In the industrial context, AI uses advanced algorithms and machine learning techniques to analyze large volumes of data, identify patterns, make predictions, and optimize processes [45].

The integration of AI into digital twins is essential to maximize their usefulness in the context of underground mining. AI allows digital twins to evolve from simple static models to intelligent systems, capable of adapting, predicting, and improving mining operations in real time [46].

Some of the applications of AI in digital twins for underground mining are the following [47]:

- Equipment fault prediction and detection: AI analyzes operational data from heavy machinery collected by sensors to identify patterns that indicate potential failures before they occur. This facilitates predictive maintenance and reduces downtime, improving overall productivity.
- Resource and operations optimization: ML algorithms process energy consumption, ventilation, and production data to optimize resource use. For example, they can automatically adjust airflows in mines to balance safety and energy efficiency.
- Predictive simulations: AI allows multiple operational scenarios to be simulated in the digital twin, such as changes in extraction methods or haulage configurations. These simulations help evaluate the impact of decisions before implementing them in the physical system, reducing risks and costs.
- Risk management: By combining real-time data and predictive modeling techniques, AI-enabled digital twins can identify hazardous conditions, such as geotechnical instabilities, before they become critical issues. This significantly improves safety in the underground environment.
- Automation and intelligent decision-making: AI helps automate complex decisions, such as equipment scheduling or haulage route optimization, based on real-time operational data and conditions.

The combination of AI and digital twins enables underground mining operations to have the following benefits: (i) reduce operational and environmental risks, (ii) maximize productivity through process optimization, (iii) improve sustainability through efficient use of resources, and (iv) increase the resilience of systems to changes in operating conditions [48].

Despite its benefits, implementing AI in digital twins faces limitations and challenges such as data quality, system interoperability, and high initial costs of technological integration.

#### 3.1.5. Machine Learning

ML is a branch of AI that focuses on the development of algorithms and models that enable systems to automatically learn and improve from data, without the need for explicit programming. These algorithms identify patterns in large volumes of data and make predictions or decisions based on them [49].

In the mining industry, ML is used to optimize processes, predict failures, analyze data in real time, and automate complex tasks that previously required human supervision.

Some applications of ML in digital twins for underground mining are as follows [50]:

- Mining operation optimization: ML algorithms analyze real-time data from operations to identify patterns and trends. This allows for the optimization of processes such as underground ventilation, material transportation, and resource distribution, reducing costs and improving operational efficiency.
- Predictive maintenance: ML is key to predictive models in digital twins, which analyze the condition of mining equipment. By detecting anomalies in operational data, digital twins can predict failures and schedule preventive maintenance, minimizing downtime and extending asset lifespan.
- Geotechnical management: In underground mining, ML enables the analysis of sensor-collected data on deformations, vibrations, and geotechnical stability. Digital twins, powered by ML, identify conditions that could pose risks, offering automated solutions to mitigate problems before they occur.
- Simulation and operational planning: Using ML, digital twins can simulate complex operational scenarios based on vast amounts of historical and real-time data. For example, they can assess the impact of an extraction design before its physical implementation, minimizing risks and maximizing productivity.
- Risk and safety analysis: By analyzing historical and current data, ML algorithms can predict dangerous situations, such as toxic gas concentrations or structural collapses, alerting operators in real time to take preventive measures.

The use of ML in digital twins brings significant benefits, such as (i) data-driven decision-making, which provides valuable insights to improve operational efficiency and safety, (ii) risk reduction, which identifies hazardous conditions before accidents occur, (iii) automation, which facilitates autonomous process management, reducing human intervention in hostile environments, and (iv) cost savings: It optimizes resources and minimizes equipment and system downtime [50].

Despite its potential, implementing ML in digital twins faces limitations and challenges, such as the need for high-quality data, model training in specific underground mining environments, and interoperability with existing systems.

#### 3.1.6. Cloud Computing

Cloud computing is a technological model that allows access to computing resources, such as servers, storage, networks, databases, and software, via the internet, on demand, and on a pay-per-use basis. This approach eliminates the need for robust local infrastructure, offering scalability, flexibility, and efficiency in managing large volumes of data [51].

Some of the applications of cloud computing in digital twins for underground mining include the following [52]:

- Real-time data processing and storage: Digital twins in underground mining generate and analyze large volumes of data from IoT sensors and other sources. Cloud computing provides the massive storage capacity and processing power needed to handle this data in real time, facilitating the generation of updated models of the physical environment.
- Distributed system integration: Cloud computing enables the integration of data from multiple locations and systems, such as geotechnical sensors, ventilation equipment, and transportation systems. This creates a centralized platform where digital twins can access all the information relevant to their operations.
- Scalable simulation and analysis: Cloud platforms offer advanced simulation and analysis tools, enabling digital twins to run complex calculations and model operational scenarios. For example, extreme geotechnical conditions can be simulated to predict risks or evaluate extraction designs before their physical implementation.
- Remote access and collaboration: Thanks to cloud computing, digital twin data and models are accessible from any location and any internet-connected device. This allows distributed teams to collaborate in real time, optimizing decision-making, even when operators are not physically at the underground mine.
- Predictive maintenance and automation: Using cloud platforms, digital twins can collect and analyze historical and real-time data to predict mining equipment failures. Additionally, the cloud facilitates connection to automation systems, improving response to critical underground mine conditions.

Cloud computing in digital twins offers some advantages, such as (i) scalability, dynamically adjusting resources according to the needs of the digital twin, such as the increase in data or the complexity of simulations, (ii) cost reduction, eliminating the need to maintain high-cost local infrastructures and allowing mining companies to optimize their technological investments, and (iii) security and backup, offering advanced data protection and redundancy mechanisms that guarantee the continuity of operations even in the face of technical failures [53].

Despite its benefits, the use of cloud computing in digital twins faces limitations and challenges, such as (i) latency in data transmission in remote locations, (ii) dependence on stable and high-speed internet connectivity in underground areas, and (iii) concerns about the security of sensitive data.

#### 3.1.7. Big Data

The term Big Data refers to the management, processing, and analysis of massive, diverse, and complex data sets that cannot be managed with traditional tools due to their volume, velocity, and variety. These characteristics, known as the “3Vs” of Big Data, have been extended to include veracity and value, highlighting the importance of reliable and relevant data for decision-making [54].

Some of the applications of Big Data in digital twins for underground mining are as follows [55,56]:

- Real-time data integration and management: In underground mining, digital twins rely on a continuous stream of data from IoT sensors, geotechnical monitoring systems, ventilation equipment, and heavy machinery. Big Data facilitates the integration and management of this data, enabling efficient, real-time processing and ensuring an accurate virtual representation of the physical environment.
- Predictive analytics and anomaly detection: Advanced Big Data analytics helps identify hidden patterns in historical and real-time data. This enables digital twins to predict equipment failures, detect anomalies in field conditions, and anticipate risks, improving safety and optimizing operations.
- Process simulation and optimization: Digital twins use Big Data to run complex simulations that evaluate extraction, geotechnical stability, or ventilation design scenarios. This helps optimize operational processes and reduce costs by minimizing planning errors.
- Environmental monitoring and sustainability: In the context of sustainability, Big Data enables the monitoring of environmental parameters such as gas levels, energy consumption, and waste generation. Digital twins can use this information to implement cleaner mining strategies and comply with environmental regulations.
- Data-driven decision-making: The use of Big Data enhances the ability of digital twins to act as decision-support tools, providing operators with detailed, real-time information about mining operations. This is essential for mitigating risks and increasing productivity.

On the other hand, among the benefits of using Big Data in digital twins, it is possible to find the following: (i) Greater precision: the availability of large volumes of data improves the accuracy of virtual models. (ii) Informed decision-making: operators have access to valuable information to respond proactively to changes in the underground environment. (iii) Resource optimization: data helps identify operational inefficiencies, allowing for more effective resource allocation [54].

The use of Big Data in digital twins faces challenges such as (i) processing capabilities, as underground mining generates large amounts of data, and analyzing it in real time requires advanced infrastructure, (ii) limited connectivity, as transmitting large volumes of data in deep underground environments can be complicated, and (iii) data security, as protecting information from cyberattacks is essential to avoid operational disruptions.

#### 3.1.8. Robotics

Robotics is a branch of engineering and technology that focuses on the design, construction, operation, and application of robots. Robots are automated, programmable, and often autonomous or semi-autonomous systems that can perform specific tasks in various environments. In the industrial context, robotics is key to optimizing processes, reducing risks, and increasing operational efficiency [57].

Some applications of robotics in digital twins for underground mining are the following [58]:

- Automation of critical operations: In underground mining, robots play an essential role in performing dangerous tasks such as drilling, loading, and hauling materials. Digital twins integrate these robots as virtual representations that replicate their movements and states in real time, enabling more precise control and monitoring.
- Inspection and monitoring in high-risk areas: Mobile robots equipped with cameras, sensors, and Light Detection and Ranging (LiDAR) systems can operate in hazardous areas, such as unstable tunnels or zones with high levels of toxic gases. Digital twins use the data generated by these robots to update virtual models, facilitating remote assessment of critical conditions.
- Robotic process optimization through simulation: Digital twins allow robot movements and operations to be simulated and tested in a virtual environment before being deployed in the physical field. This minimizes errors and downtime, increasing operational efficiency.
- Autonomous and predictive maintenance: Maintenance robots, such as those equipped with manipulator arms or drones, can inspect equipment and perform minor repairs. Digital twins integrate the data obtained by these robots to predict failures and plan interventions before serious breakdowns occur.
- Coordination of autonomous teams: Digital twins enable the coordination of multiple autonomous robots in underground environments. For example, material transport routes can be optimized or drilling and loading tasks synchronized to maximize productivity.

On the other hand, some of the benefits of using robotics in digital twins are the following: (i) Increased safety: robots reduce workers’ exposure to hazardous environments. (ii) Operational efficiency: robotic operations, combined with digital twins, maximize productive time by optimizing processes. (iii) Precision in control: virtual models allow detailed monitoring and adjust robotic operations in real time.

In addition, it is possible to mention that there are challenges in the implementation of this technology, such as the following: (i) Need for robust infrastructure: robotics in underground mining requires reliable communication systems to interact with digital twins. (ii) High initial costs: the development and integration of robots in digital twins can be expensive. (iii) Requirement for staff training: it is necessary to train operators to work with robots and advanced technologies.

Robotics, integrated with digital twins, represents a transformative solution for addressing the challenges of underground mining. By enabling automation, simulation, and remote monitoring, this technological combination significantly improves safety, productivity, and sustainability in mining operations.

#### 3.1.9. Automation

Automation refers to the use of technological systems to perform tasks with minimal or no human intervention. This technology employs tools such as sensors, actuators, advanced software, and control systems to improve efficiency, precision, and safety in industrial processes. In mining, automation has evolved toward a more advanced approach, incorporating artificial intelligence, robotics, and autonomous systems [16].

Some of the applications of automation in digital twins for underground mining are as follows [16]:

- Autonomous control of machinery and equipment: Automation in underground mining applies to equipment such as drills, loaders, and autonomous trucks, allowing them to operate without direct intervention. Digital twins replicate these automated systems in a virtual environment, allowing operators to monitor performance, analyze data in real time, and optimize processes.
- Mining operation optimization: Automated systems, integrated with digital twins, can plan and coordinate activities such as drilling, blasting, and material hauling. This ensures a continuous and efficient workflow, minimizing disruptions and downtime.
- Dynamic monitoring and adjustment: Digital twins use data generated by automated systems to model and simulate different operating scenarios. This allows parameters to be adjusted in real time to maximize safety and productivity in complex underground conditions.
- Predictive maintenance automation: Integrating automation with digital twins allows equipment failures to be predicted using predictive analytics algorithms. Systems can automatically schedule machine maintenance before critical failures occur, reducing costs and improving reliability.
- Automated ventilation systems: in underground environments, automated ventilation systems, linked to digital twins, optimize airflow based on the specific needs of each operation, improving environmental conditions and reducing energy consumption.

On the other hand, the benefits of automation integrated with digital twins are the following: (i) Increased safety: it reduces the exposure of workers to hazardous areas. (ii) Increased productivity: automated operations are faster and more precise. (iii) Resource optimization: systems automatically adjust the use of materials and energy according to needs. (iv) Data-driven decision-making: digital twins provide a complete analysis of automated operations [59].

The implementation of automation and digital twins has inherent challenges, which are as follows: (i) High initial costs: the implementation of automated technology and its integration with digital twins requires a significant investment. (ii) Personnel training: workers need to be trained to manage and operate advanced automated systems. (iii) Connectivity limitations: in deep mines, ensuring communication between automated systems and digital twins can be a technical challenge [59].

Automation, integrated with digital twins, radically transforms underground mining operations. By enabling precise control, real-time monitoring, and continuous optimization, this technological combination not only improves productivity and safety but also sets a new standard for sustainability in the mining industry.

### 3.2. Components of Underground Mine Digital Twins

The following components of an underground mine digital twin are presented below: (i) physical entity, (ii) data collection system, (iii) data connectivity and data storage/processing system, (iv) virtual models, and (v) users. These will be explained in detail in the following paragraphs.

#### 3.2.1. Physical Entity (Underground Mine)

A physical entity is the tangible element of the real world that is replicated in the digital environment. It is the foundation upon which the digital twin is built and can range from simple objects to complex systems, in this case, the underground mine. Physical entity must have quantifiable parameters through sensors, such as (i) physical variables, including temperature, pressure, vibration, and humidity; (ii) operational variables, including energy consumption and efficiency; and (iii) state variables, including wear, failures, and life cycles, among others [60,61].

The digital twin’s interaction with the environment is relevant, as it can receive and respond to external influences (e.g., ventilation conditions, mining truck traffic, etc.), which the digital twin must reflect [6].

Considering physical entity, the following factors are important in the digital twin [6]:

- Replication accuracy: the better the physical entity is monitored, the more accurate the digital twin will be.
- Scalability: some digital twins can integrate multiple interconnected physical entities (e.g., a multi-level underground mine connected to conveyor belt extraction systems).
- Full lifecycle: the digital twin can follow the physical entity from design to obsolescence, enabling continuous optimizations.

Finally, some challenges and issues regarding the physical entity in the digital twin are as follows: (i) Instrumentation: not all physical entities have integrated sensors (e.g., old infrastructures), which require adaptations. (ii) Dynamism: some entities change rapidly (e.g., moving machinery and changing blast plans), demanding real-time updates.

#### 3.2.2. Data Collection System (Sensors)

The data collection system is the component responsible for capturing real-time information from the physical twin to feed into the virtual model. Its efficiency determines the accuracy and usefulness of the digital twin. It corresponds to the set of sensors, IoT devices, and tools that capture real-time data from the physical entity, such as temperature, vibration, pressure, location, performance, etc. [62,63].

The components of the data collection system are as follows [64,65]:

- IoT sensors and devices: (i) Physical sensors measure variables such as temperature, pressure, vibration, humidity, electric current, etc. (ii) IoT (Internet of Things) devices allow wireless data transmission. (iii) Actuators not only capture data but can also interact with the physical system (feedback).
- Acquisition hardware (DAQ): (i) data acquisition cards (e.g., NI DAQ from National Instruments), (ii) PLCs (Programmable Logic Controllers) in industrial environments, and (iii) embedded systems (Raspberry Pi, Arduino) for prototypes.
- Capture and preprocessing software: (i) Firmware on devices: initial processing of data before sending it. (ii) Gateways IoT: filter and compress data before sending it to the cloud. (iii) Edge computing: local processing to reduce latency (e.g.: Azure IoT Edge, AWS Greengrass).

Regarding the challenges in data collection, the following are considered: (i) data quality as noise in the signals, which are filtered with algorithms (Kalman Filter, wavelet transform), and incomplete data, which requires imputation techniques (interpolation, generative AI); (ii) scalability and bandwidth—in systems with thousands of sensors, efficient protocols such as LoRaWAN are used; (iii) security and privacy, requiring data encryption (TLS/SSL) and Blockchain for traceability in critical environments; and (iv) time synchronization, for which protocols such as IEEE 1588 (PTP—Precision Time Protocol) are used to avoid offsets.

Finally, as future trends, we have self-powered sensors (energy harvesting), 5G/6G networks for low latency in real time, and cognitive digital twins (AI that learns from physical behavior).

#### 3.2.3. Data Connectivity and Data Storage/Processing System (IoT, Cloud Computing, and AI)

The data connectivity and processing system is a critical component of a digital twin, as it acts as the “nervous system” that links the physical entity to its virtual representation. It is the infrastructure that allows the collected data to be transmitted, stored, and processed. It includes communication networks (5G, IoT, edge), computing databases, and algorithms to filter, analyze, and prepare information for use in the virtual model [5].

Below, we delve into its architecture, key technologies, and features [66,67]:

- Main functions: (i) data transmission: connects sensors and IoT devices with processing platforms, where they guarantee low latency and high reliability (e.g., for real-time industrial applications).
- Processing and storage: filters, cleans, and structures raw data (e.g., removing noise from vibration signals), stores historical data for trend analysis and AI model training.
- Integration with virtual models: feed processed data into simulation models, dashboards, or prediction algorithms.

#### 3.2.4. Virtual Models

Virtual models are the core of a digital twin, as they replicate the behavior, characteristics, and dynamics of the physical entity in a computational environment. Their complexity can range from simple geometric representations to advanced simulation systems powered by artificial intelligence. Therefore, they are the computational representation of the physical entity, which can include simulations, 3D models, AI/ML algorithms, and prediction systems. These models are updated with data in real time to reflect the state and behavior of the physical twin [6,7].

**Figure 5:**
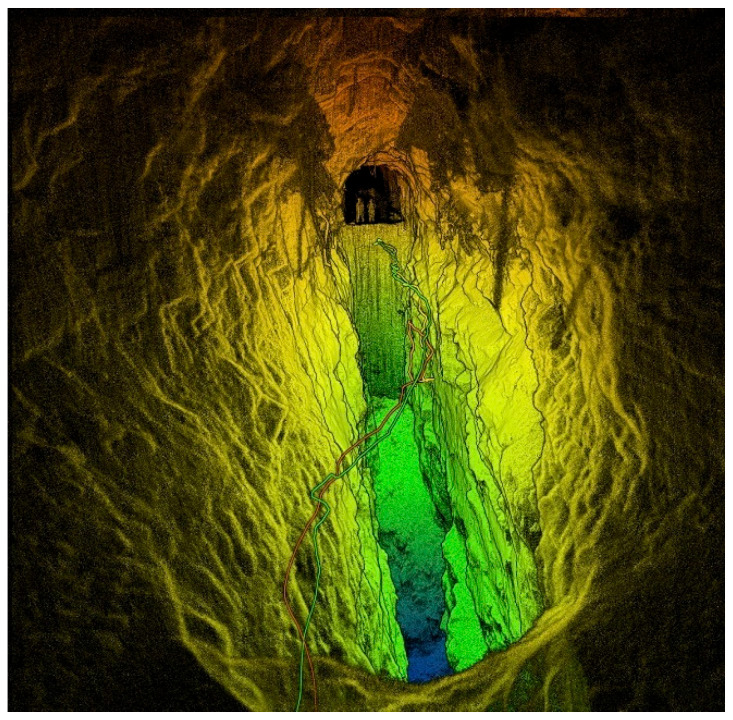
Example of geometric model (CAD/3D 2025 version).

**Figure 6:**
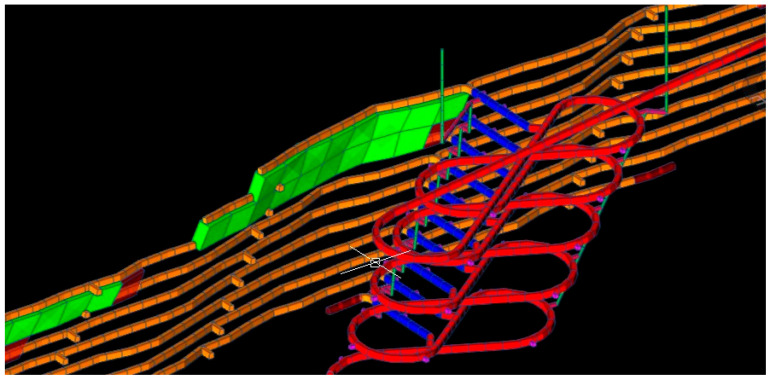
Example of physical–mathematical models of geological–mining planning with software Datamine 2025 version.

**Figure 7:**
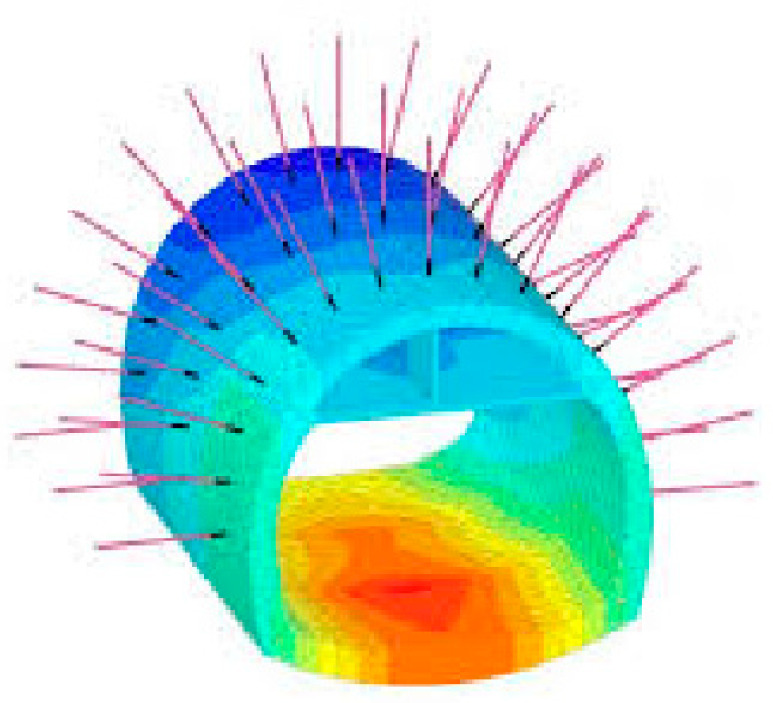
Example of a dynamic simulation and finite element model (FEM) with the software PLAXIS 2025 version.

The most common type of virtual models for underground mines are the following:

- Geometric models (CAD/3D): 3D visual representation of the physical entity, created with tools such as CAD (Computer-Aided Design) (see Figure 5), where the main uses are the following: (i) visualization of the physical asset; (ii) design and ergonomics analysis, integration of the use of drones with LiDAR technology, RGB images with the use of photogrammetry, and use with AR; (iii) and VR.

- Physical–mathematical models for geological–mining planning: based on equations that describe system behavior (rock mechanics, mining plan, ventilation, etc.) (see Figure 6). Some examples of software packages include Surpac, Vulcan, Datamine, Minesight, and Promine, among others.

- Dynamic simulation and finite element models (FEMs): It is possible to predict the behavior of the system in different operating conditions (see Figure 7), where the common tools are the following: (i) MATLAB Simulink (control systems), (ii) ANSYS (multidisciplinary engineering), and (iii) Modelica (modeling of complex physical systems). Some other examples of software packages include COMSOL, FLAC3D, PLAXIS, and MIDAS GTS NX.

- Data-driven models: they use ML and AI to learn patterns from historical and real-time data, where some examples are the following: (i) neural networks to predict machinery failures and (ii) clustering algorithms to optimize industrial processes.
- Hybrid models (physical + data-driven): they combine physics-based models with AI techniques for greater accuracy, where they are useful when pure equations do not capture all the complexity of the system.

Some challenges in the development of virtual models are the following: (i) Computational complexity: very detailed models require high processing power. (ii) Data quality: if the sensors fail, the model loses accuracy. (iii) Integration of multiple disciplines: a digital twin may require experts in mechanics, software, and data. (iv) Security and cybersecurity: there is a risk of attacks on critical connected systems.

#### 3.2.5. User

Users are a key component of the digital twin ecosystem, as they interact with virtual representations to make decisions, optimize processes, or improve the performance of the physical system. Users are the people or systems that interact with the digital twin to monitor, analyze, optimize, or make decisions. They can be engineers, operators, managers, or even automated control systems [12,68].

The following details the types of users, their roles, and how they interact with the digital twin.

- Operators and technicians: (i) Role: real-time monitoring and maintenance. (ii) Interaction: view alerts and anomalies (e.g., excessive vibrations in a machine), receive predictive maintenance instructions, and use simple interfaces (dashboards, augmented reality).
- Engineers and designers: (i) Role: continuous optimization and improvement. (ii) Interaction: analyze historical data and simulations to redesign components, test “ what-if “ scenarios before implementing changes in the real world, and use advanced tools (CAD, FEM simulators, machine learning models).
- Managers and decision-makers: (i) Role: strategy and resource management. (ii) Interaction: analyze KPIs (performance, costs, energy efficiency), make decisions based on projections (e.g., when to replace an asset), use dashboards with intuitive visualizations (charts, control panels).
- Automated systems (AI/ML and controllers): (i) Role: autonomy and real-time adjustment. (ii) Interaction: AI algorithms that adjust parameters automatically (e.g., smart ventilation system). (iii) Control systems that react to changes without human intervention.

Some benefits for users when using an underground mine digital twin are (i) error reduction, requiring less manual intervention thanks to pre-simulation, (ii) faster decision-making, featuring access to real-time data and predictions, (iii) improved training, i.e., training in virtual environments before operating real equipment, and (iv) cost optimization, such as the early detection of faults and better use of resources.

Finally, some challenges in the user experience are (i) information overload, as too much data can overwhelm operators, (ii) complex interfaces, as not all users have advanced technical skills, and (iii) security and privacy, calling for differentiated access to avoid leaks or manipulations.

## 4. Advanced Multilayer Conceptual Framework of Underground Mine Digital Twins

**Figure 8:**
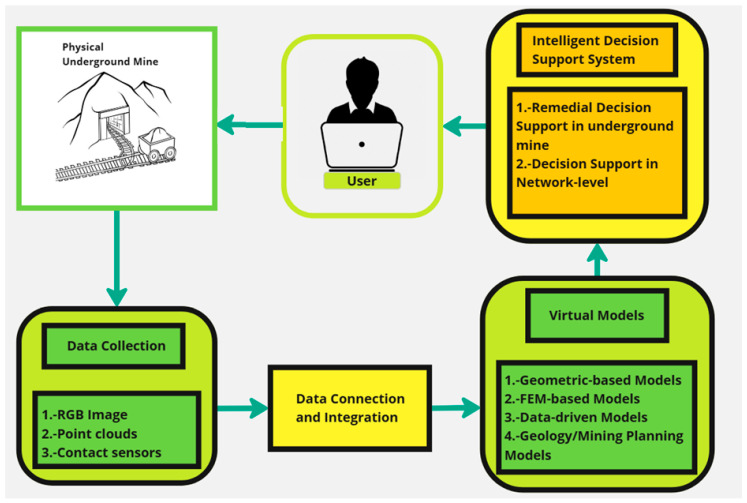
Simplified diagram of the advanced multi-layer digital twin conceptual framework.

A simplified diagram of the advanced multi-layer digital twin conceptual framework is presented in Figure 8, where each of its components is shown in a simplified and summarized manner: (i) physical entity, (ii) data collection system, (iii) data connection and integration system, (iv) virtual models, (v) intelligent decision support system, and (vi) user.

Figure 8 represents a flowchart describing the transfer of information in a digital twin model for underground mining. The diagram shows the flow of information from the physical mine to a decision support system, through data collection and the creation of virtual models. User feedback is included.

The process begins in a physical underground mine, represented by a schematic drawing of a mine with a mine car on rails. Data is collected from the underground mine, represented by the “Data Collection” block, which includes RGB images, point clouds, and contact sensor data. This information is integrated through the “Data Connection and Integration” block and used to create virtual models. Connection systems can include 3G/4G/5G cellular networks, LoRaWAN systems, and wired systems, among others.

Virtual models are divided into four types: geometry-based models, finite element models (FEMs), data-driven models, and geological/mine planning models. The virtual models are then fed into an intelligent decision support system, which provides support for remediation decisions in the underground mine and for network-level decision-making. Finally, a user interacts with the system via a laptop.

In summary, Figure 8 illustrates a system that connects real-world (physical mine) data with virtual models to support decision-making in underground mine management, providing a tool for more efficient and safe mining.

**Figure 9:**
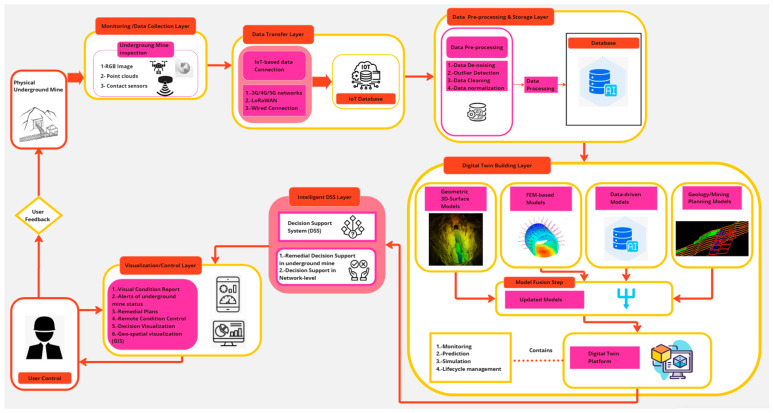
Detailed diagram of advanced multi-layered digital twin conceptual framework of underground mine.

The advanced multilayer conceptual framework of the underground mine digital twin is presented in the Figure 9, where each of its components is shown in detail: (i) physical entity, (ii) monitoring/data collection layer, (iii) data transfer layer, (iv) data preprocessing and storage layer, (v) digital twin construction layer, (vi) intelligent decision support system (DSS) layer, (vii) visualization/control layer, and (viii) user feedback.

Figure 9 presents a flowchart describing the architecture of a digital twin system for underground mines. The diagram is divided into several layers, interconnected to show the data flow and the digital twin creation process.

The following explains in detail each of the layers that make up the advanced multi-layered conceptual framework for an underground mine digital twin:

1. Physical entity: underground mine.
2. Monitoring/data collection layer: This layer starts with the physical underground mine as the data source. Large volumes of data are collected through different types of sensors: RGB images, point clouds, and contact sensors.

The monitoring and data collection layer constitutes the sensory foundation of the digital twin and is fundamentally reactive, designed to capture the physical state of the mine in real or near real time. This layer begins with the physical underground mine as the primary source of data, a dynamic and constantly changing environment due to excavation, drilling, blasting, and the movement of personnel and machinery. Its main function is to continuously digitize this physical environment, translating real-world conditions and events into a constant stream of digital data. Without this layer, the digital twin would lack the raw material necessary to represent, analyze, or simulate reality, becoming a static and obsolete model.

The collection of these large volumes of data is achieved through a heterogeneous and multimodal network of sensors, each capturing a specific type of information. RGB images from cameras distributed throughout drifts, work faces, and machinery provide immediate visual context for monitoring operations, personnel safety, and verifying procedures. On the other hand, technologies such as LiDAR and laser scanners generate three-dimensional point clouds, which are essential for capturing tunnel geometry, excavation profiles, and extracted material volumes with high precision, as well as detecting deformations or displacements in mine walls and ceilings. Finally, contact sensors and other types of wireless (IoT) sensors measure critical physical parameters such as seismic vibrations following a blast, concentrations of toxic or explosive gases (methane, carbon monoxide), air quality, structural stability in supports, and the operational status of critical equipment such as pumps, fans, and conveyor belts.

Together, this layer functions as the nervous system of the digital twin. The synergy between the different types of data is crucial; for example, a point cloud can show a change in the geometry of a tunnel, while vibration sensors can confirm that it was caused by a nearby blast, and RGB images can verify that the area was clear at the time of the event. This massive and diverse collection of raw data lays the foundation for the upper layers of the twin, where information will be integrated, processed, analyzed, and used for visualization, simulation, and proactive decision-making.

3. Data transfer layer: This layer is responsible for the connection between the monitoring layer and the preprocessing and storage layer. This information is transmitted through different networks (3G/4G/5G, LoRaWAN, wired connection, among others) to an IoT database.
4. Data preprocessing and storage layer: The collected data is preprocessed to improve its quality and usefulness. This process includes noise removal, outlier detection, data cleaning, and normalization. The processed data is stored in a database which incorporates artificial intelligence (AI) for analysis.

The data transfer layer acts as the digital twin’s circulatory system, solely responsible for the secure and efficient transport of raw data from the mine’s monitoring devices to the preprocessing and storage systems. Its primary function is to establish a reliable communication bridge across the hostile and complex underground environment, overcoming challenges such as signal attenuation, interference, and lack of infrastructure. This layer does not process or modify the data; its critical mission is to guarantee its integrity and availability, ensuring that every bit of information captured by the sensors reaches its destination for subsequent analysis, without becoming a bottleneck for the real-time system.

The choice of network technology on this layer is not unique but rather responds to a hybrid and multifaceted strategy designed for the specific needs of underground mining. For the transmission of large volumes of high-speed data, such as real-time video from cameras or laser scanning of tunnels, wired connections (fiber optic or Ethernet) are prioritized in key areas, offering maximum bandwidth and stability. In tunnels and mobile work faces, where cables are unfeasible, wireless networks such as 5G are deployed, which provide low latency and high capacity, or long-range, low-power networks such as LoRaWAN, ideal for telemetry from thousands of dispersed sensors (such as air quality or geotechnical displacement sensors) that send small data packets intermittently and with minimal power consumption.

Ultimately, the destination of all these data streams is a centralized IoT database, which serves as the consolidation point for information. This database is optimized to handle massive time series, continuously ingesting telemetry, location, and status data from all networks. The robustness of the data transfer layer is therefore critical: any communication failure would result in information gaps in the digital twin, compromising its accuracy and its ability to provide a reliable representation of the current state of the mine. Thus, this layer lays the foundation for all the analysis, simulation, and decision-making operations that will be built into the upper layers of the digital twin.

5. Digital twin construction layer: This layer uses processed data to create various models: 3D geometric surface models, finite element models (FEM), data-driven models (using AI), and geological/mine planning models. These models are then merged to generate updated models that form the digital twin. This layer also contains the core functionalities of the digital twin platform, such as monitoring, prediction, simulation, and lifecycle management.

The model fusion layer is the intelligent core of a mining digital twin. Its main function is to integrate all the mine’s dispersed information—such as 3D plans, geological data, sensor readings, and equipment locations—into a single, unified virtual model. It does not simply combine data; it synchronizes it into a single coordinate system and timeline, creating a coherent and contextualized digital replica of the physical mine.

This layer acts as the “brain” that gives meaning to the data. By correlating information in real time, it goes beyond visualization to establish causal relationships. For example, it can overlay the exact location of a loader with the ore grade model, or cross-reference seismicity data with a tunnel’s structural model to assess stability risks. This contextualization is what transforms the data into actionable information.

Thanks to this fusion, the digital twin becomes a powerful tool for decision-making. It allows for simulating scenarios, optimizing live operations, predicting equipment failures, and generating proactive safety alerts. Essentially, this layer is what differentiates a simple 3D model from an operational intelligence system that can transform the efficiency and safety of an underground mine.

6. Intelligent decision support system (DSS) layer: This layer is crucial for data analysis. The decision support system (DSS) provides two important functions: decision support for corrective measures in the underground mine and decision support at the network level.

The intelligent decision support system (DSS) layer acts as the cognitive hub of the digital twin, transforming raw data into actionable knowledge. In the context of underground mine decision-making, this system integrates and analyzes multidisciplinary information from sensors, equipment, and geological models in real time. For example, it can correlate data on rock stability, ventilation levels, personnel location, and drilling rig performance. Using artificial intelligence and machine learning techniques, the DSS can simulate scenarios, predict critical events such as structural failures or gas accumulations, and recommend specific actions. This allows mine supervisors to optimize the extraction sequence, proactively manage safety risks, and adjust logistics operations instantly, ensuring more efficient and safe production.

Simultaneously, the network-level decision support function elevates the perspective from the individual mine to the entire operational value chain. At this level, the DSS analyzes the impact of underground operations on downstream processes, such as transportation, processing at the concentrator plant, shipping logistics, and inventory management. The system can model how a delay at a specific production front will affect the consistent supply of ore to the plant, or how a variation in ore grade requires adjustments to grinding and flotation parameters. By holistically optimizing the entire network, the DSS helps maximize overall business performance, reduce bottlenecks, minimize integrated operating costs, and ensure delivery commitments to customers are met, based on a unified, real-time view of material and information flow.

The synergy between these two functions—local operational and network strategic—is what gives the digital twin its truly transformative power. The DSS not only solves isolated problems but creates a continuous cycle of improvement. A decision made at the mine level is immediately evaluated in terms of its impact on the network. Conversely, strategic production or quality objectives dictated by management are translated into precise operational guidelines for the subsurface. This layer, therefore, transcends mere automation to become an intelligent partner for management, facilitating data-driven, predictive decision-making that optimally balances safety, operational efficiency, and profitability criteria in a dynamic and complex environment.

7. Visualization/control layer: This layer provides a user interface for visualizing the status of the underground mine, including visual condition reports, alerts, remediation plans, remote condition control, decision visualization, and geospatial visualization (GIS). A representative user interface with graphics and visualizations is shown. For example, results can be viewed through smartphones, tablets, or computer screens.
8. User feedback: a feedback loop is included that allows the user to interact with the system and provide information that will be used to improve the process.

Finally, the integration of all these layers and the model fusion stage creates the digital twin platform used for monitoring, prediction, simulation, and lifecycle management of the underground mine.

In summary, Figure 9 represents the advanced multi-layered conceptual framework for an underground mine digital twin, a complete and integrated system for monitoring, analyzing, and managing underground mines through a digital twin platform. The image highlights the importance of data collection, data processing, data analysis with artificial intelligence, and visualization for informed decision-making in mine management.

## 5. Functionalities of the Underground Mine Advanced Multi-Layered Digital Twin Conceptual Framework

The following section presents each of the functionalities of the advanced, multi-layered conceptual framework for a digital twin of an underground mine: (i) real-time monitoring, (ii) anomaly prediction, (iii) scenario simulation, (iv) lifecycle management, and (v) decision support system.

### 5.1. Monitoring in Real Time

Real-time monitoring functionality in an underground mine’s digital twin consists of the ability to collect, process, and visualize continuously updated data to reflect the operational status, environmental conditions, and performance of mine equipment. This capability is key to proactive decision-making and operational optimization.

Some key benefits of real-time monitoring in underground mine digital twins are (i) a reduction in emergency response times, (ii) increased productivity through data-driven decisions, (iii) greater job safety by anticipating risks, and (iv) cost optimization through preventive maintenance.

This functionality makes the digital twin an essential tool for Mining 4.0, enabling smarter and safer operations.

### 5.2. Prediction of Anomalies

An anomaly prediction in an underground mine’s digital twin is an advanced capability that uses analytical models, AI, and real-time data to anticipate failures, operational risks, or hazardous conditions before they occur.

Predictive modeling employs ML algorithms and statistical models to identify patterns associated with past failures (e.g., collapses, equipment failures, gas leaks). It uses techniques such as (i) neural networks to predict complex anomalies, (ii) time series (e.g., ARIMA, Prophet) to anticipate critical trends, and (iii) outlier detection (e.g., Isolation Forest, SVM) in operational data. In addition, it is possible to define early warnings where (i) automatic notifications are generated when indicators exceed safe thresholds (e.g., methane concentration, tunnel deformation) and (ii) anomalies are classified by severity (mild, critical, emergency).

Some benefits of anomaly prediction are (i) a reduction in accidents (e.g., collapses, explosions), (ii) cost optimization by avoiding unplanned shutdowns, and (iii) regulatory compliance through proactive risk control.

### 5.3. Simulation of Scenarios

Scenario simulation functionality in a digital twin of an underground mine is a key capability that enables modeling, analyzing, and predicting mining system behavior under different operating, environmental, or design conditions. This functionality uses real-time data, physical and mathematical models, and artificial intelligence algorithms to recreate virtual situations that aid in decision-making.

Scenario simulation is feasible where (i) the potential impact of an anomaly is evaluated using physics-based simulations (e.g., structural stability, ventilation flow) and (ii) it allows corrective measures to be tested in the virtual environment before applying them in the real mine.

For example, simulation of mining operations is developed as follows: (i) modeling of processes such as drilling, blasting, loading, transport, and ventilation, (ii) optimization of equipment routes (LHD, trucks) to improve productivity, and (iii) evaluation of production scenarios under different extraction rates.

Some of the benefits associated with this functionality are the following: (i) cost reduction, as it avoids expensive physical tests, (ii) greater security, as it identifies risks before implementing changes, (iii) continuous optimization, as it adjusts operations based on predictive data, and (iv) sustainability, as it minimizes environmental impacts through advanced modeling.

### 5.4. Lifecycle Management

Lifecycle management (LCM) of an underground mine’s digital twin involves controlling and optimizing all stages of the twin, from its creation to its update and eventual retirement, integrating real-time data, predictive models, and advanced analytics to improve mining operations.

It is possible to carry out maintenance and optimization activities that consider (i) Automatic alerts for deviations from safety parameters, (ii) predictive maintenance planning of equipment, and (iii) process adjustment based on historical and live data.

In addition, it is possible to develop an update and evolution of the digital twin according to the following characteristics: (i) ML to improve models with new data and (ii) adaptation to changes in exploitation (new veins, expansions).

Finally, it is feasible to execute the retirement or migration phases considering the following key aspects: (i) archiving historical data for future analysis and (ii) transition to new models when the mine closes or is modernized.

Some of the benefits of an efficient LCM are (i) risk reduction (operational safety), (ii) increased productivity (better extraction planning), (iii) sustainability (energy and resource optimization), and (iv) data-driven decisions (avoids unplanned shutdowns).

A robust LCM digital twin enables smarter, safer, and more efficient underground mine operations.

### 5.5. Decision Support System

A decision support system (DSS) for an underground mine digital twin is a technological tool that integrates real-time data, predictive models, and simulations to optimize decision-making in the operation and management of an underground mine. Its core functionality focuses on improving efficiency, safety, and profitability through data analysis and interactive visualization.

Furthermore, it is an integrated technology platform that combines dynamic digital models, real-time data, advanced analytics, and simulation tools to assist in operational, strategic, and tactical decision-making in underground mining.

Some enabling technologies of the decision support system are (i) cloud computing (Big Data processing), (ii) edge computing (field analysis for low latency), (iii) AI/ML (neural networks for geotechnical patterns), and (iv) blockchain (mineral traceability and smart contracts) [51,69].

Finally, it is possible to highlight some benefits, such as (i) cost reduction through resource optimization, (ii) greater security with early risk warnings, (iii) decision-making based on data instead of intuition, and (iv) sustainability through better energy and waste management.

## 6. Validation by a Panel of Experts

A questionnaire was designed using the Delphi method, adapted to achieve consensus among experts on specific technical topics. The 15 statements comprising the instrument were generated from an exhaustive literature review and preliminary discussions with a small group of specialists, thus ensuring their relevance and content validity. Each statement was structured as a closed-ended question, predominantly using a five-point Likert scale ranging from “Strongly disagree” to “Strongly agree.” This approach not only facilitates the quantification of perceptions and the statistical analysis of the data but also reduces ambiguity in responses, making the process more efficient for participants, who are professionals with time constraints. The survey was distributed digitally, accompanied by a cover letter explaining the study’s objectives, data confidentiality, and instructions for completion.

The selection of experts was governed by two fundamental and non-exclusive criteria, designed to ensure that participants possessed in-depth and practical knowledge of the field of study. First, a minimum of more than eight years of professional experience in the mining industry was required, ensuring that participants had faced a variety of operational scenarios and possessed a consolidated perspective. Second, and crucially, direct and verifiable experience in underground mining projects was required, as the specifics of this mining method are radically different from those of open-pit mining. The final sample size was 10 experts, which is considered adequate and manageable for qualitative and consensus-building studies with expert panels, where the quality and depth of knowledge take precedence over numerical quantity. Their professional profiles are presented in Table 3:

Of the 15 mining experts identified and formally invited to participate in the study, a group of 10 accepted the invitation, resulting in a response rate of 66.7%. This rate is considered high for this type of methodology aimed at high-level professionals, reflecting the perceived interest in the topic and the effectiveness of the outreach strategy. The data collection process took place over three weeks, with periodic reminders to ensure complete responses. Participation of 100% of the selected panel (the 10 who accepted) was ultimately achieved, resulting in 10 complete and valid questionnaires for analysis.

According to Table 3 most of the experts interviewed are mining engineers from Chile and Peru, with between 8 and 25 years of experience. Some experts hold master’s degrees, MBAs, or doctoral degrees. Most of them work in the field of underground mining, specializing in areas such as design, operations, and safety.

The relationship between these statements and the literature review in this study is linked to the components and functionalities of digital twins in underground mining. The statements were formulated by considering the most relevant criteria related to operational efficiency, sustainability, and safety in underground mining operations (see Table 4).

The experts completed a questionnaire with the 15 statements defined in Table 5. The questions were answered on a 5-point Likert scale, with a minimum score of 1, meaning “strongly disagree,” and a maximum score of 5, corresponding to “strongly agree.”

**Figure 10:**
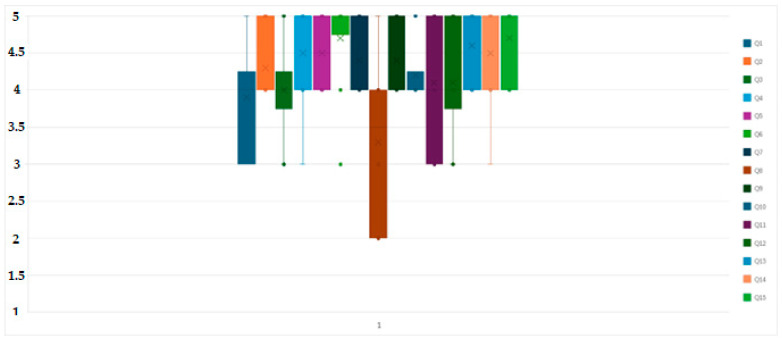
Results of questions to panel of experts.

The results of the questions to the panel of experts are shown in Figure 10:

Figure 10 shows a box and whisker plot summarizing the responses to the 15 questions posed to the expert panel. Questions 2, 4, 5, 7, 9, 13, 14, and 15 received mostly positive responses. The response to question 8 was less positive, while the responses to the remaining questions were generally somewhat positive.

The results were analyzed using two approaches: quantitative and qualitative. Quantitatively, measures of central tendency (mean, median) and dispersion (standard deviation) were calculated for each of the 15 statements to determine the level of overall agreement and consensus within the panel. For example, statements that obtained a median of 4 or 5 (on a scale of 1 to 5) and a low standard deviation were interpreted as areas of strong consensus. Qualitatively, the optional open-ended comments that experts could add were analyzed, allowing for a richer interpretation of the numerical data and an understanding of the nuances behind the assessments. Specific results showed a high degree of consensus (defined as more than 60% of responses in the “Agree” and “Strongly Agree” categories) for 8 of the 15 statements, validating the strength of most of the postulates presented. The remaining seven statements, which showed moderate dispersion, were identified as topics requiring further discussion or future research, thus providing a clear roadmap for further work.

Questions Q1 to Q14 are hypothesized (H1 to H14) and were tested using nonparametric statistical methods. This approach was chosen because the data consisted of a small sample of ordinal measurements that violated the normality assumption required for parametric tests. Nonparametric methods are well-suited for such data, as they do not assume an underlying normal distribution or require data to be on an interval or ratio scale

Each hypothesis was evaluated by comparing the observed median of its variable against the scale’s neutral point (median = 3) using a one-sample Wilcoxon signed-rank test. The tests were one-tailed, with the null hypothesis (H_0_) stating the median was ≤3 and the alternative (Hₐ) stating it was >3 (see Table 6).

All tests yielded significant results (*p*-value < 0.05), leading to the rejection of the null hypothesis and supporting the conclusion that each question’s median was indeed greater than three.

Question 15 was designed to evaluate the level of consensus among the experts’ answers. To this end, the Fleiss Kappa coefficient (ρ), which ranges from −1 to 1, was employed to all questions. A value of 1 signifies perfect agreement, while values approaching −1 indicate a complete lack of agreement; intermediate values suggest a fair to moderate consensus. The null hypothesis (H0) posited no agreement among experts, while the alternative hypothesis (HA) proposed that agreement existed. The analysis resulted in a coefficient of 0.056, which was interpreted as demonstrating a significant level of agreement.

The validity (the instrument’s accuracy in measuring its intended target) and reliability (the consistency and reproducibility of its results) of the questionnaire were subsequently evaluated. Inter-item correlations were established and examined using the Spearman’s rank correlation coefficient (ρ), which ranges from −1 to 1, with the results presented in Table 7.

The analysis revealed a significant negative correlation (ρ ≤ 0) between variables presumed to measure the same construct. This finding suggests these variables are not capturing the intended construct and should consequently be considered for removal from the instrument. The values highlighted in bold and with an asterisk indicate the questions that have the highest positive correlation according to Spearman’s correlation coefficient.

The analysis revealed no significant negative correlations (Table 7), leading to the retention of all survey items. Furthermore, the instrument’s internal consistency was validated using Cronbach’s alpha. The calculated value of 0.781 for the 14-item scale exceeded the accepted benchmark of 0.7 [70], demonstrating reliability for this type of research.

## 7. Comparative Analysis of the Theoretical Framework Defined in This Study with Existing Research

The concept of the DT in underground mining represents one of the most significant technological evolutions of Industry 4.0 for this sector. Essentially, a DT is a dynamic virtual representation of a physical asset, process, or system, synchronized through bidirectional data flows. However, there is a considerable gap between its theoretical conceptualization and its actual practical application. Academic and cutting-edge literature emphasizes bidirectionality and real-time simulation, defining the DT as a “living” cyber-physical system that learns, self-updates, and predicts future behaviors using AI and ML. It is considered the natural evolution of mine planning, BIM, and SCADA systems, forming an integrated ecosystem that can range from a twin of a specific asset (such as a shovel or fan) to a twin of the entire mining enterprise. In contrast, current practical applications in industry and commercial solutions are typically more modest and incremental. Often, what is implemented is closer to a “Visual Twin” or a data-enriched 3D digital model, where data flows are predominantly unidirectional (from the physical to the digital world) for monitoring and visualization. Implementation typically begins with twins of critical assets or specific systems, such as ventilation, based on existing foundations such as BIM models, rather than building a complete enterprise twin from scratch.

This divergence between theory and practice becomes evident when analyzing its key components. The literature idealizes a dynamic virtual model, based on physics and data, that simulates the behavior of the physical system under different scenarios. This model would be fed by a high-fidelity, real-time, bidirectional data flow, allowing not only diagnosis but also prediction and prescription of autonomous optimization actions using advanced analytics and AI. In the operational reality of an underground mine, the virtual model is typically a static or periodically updated 3D model, where simulations (of ventilation or stability, for example) are run separately and not fully integrated into a real-time cycle. Data connection is hampered by persistent connectivity challenges underground, which severely limit bidirectionality. While AI-powered predictive equipment maintenance is a mature application with a high return of investment, AI-based prescriptive analytics and comprehensive simulation are still in their infancy and require human validation.

Specific applications reflect this transition from promise to implementation. The potential described in the literature includes the simulation of multiple mining scenarios to design the optimal mine before operations begin, real-time autonomous optimization of fleet and production cycles, and the proactive prediction of safety events such as structural failures. In practice, success stories focus on discrete areas: 3D visualization for planning, fleet management systems for tracking and productivity, real-time environmental monitoring, and, above all, predictive equipment maintenance. The autonomous management of complex systems, such as ventilation that dynamically adjusts to energy consumption and mining activity, is a goal that has not yet been widely adopted. The challenges to closing this gap are formidable and include the critical lack of connectivity and robust data infrastructure in underground environments, the difficulty of integrating heterogeneous systems (an interoperability issue), the quality and standardization of data generated in the field, and the inherent uncertainty in geological modeling, which underlies the entire operation. Furthermore, literature often underestimates the cultural barrier and the need for a transformation into human capital to adopt these new paradigms.

In short, the digital twin in underground mining is in an early stage of maturity, where its implementation is incremental and focused on solving specific, high-value problems. The future trend points to an evolution from descriptive to prescriptive systems, where DT not only displays data but also recommends and executes actions. The integration of asset twins into comprehensive process twins, the use of hybrid simulations (physical and AI/ML-based), and a focus on sustainability will be key. The ultimate value of the DT will lie not in the fidelity of its visualization, but in its ability to transform data into actionable decisions that tangibly improve the safety, productivity, and sustainability of complex underground mining operations.

## 8. Discussion: Future Directions Related to Underground Mine Digital Twins

Digital twins have emerged as a transformative technology in the mining industry, enabling the virtual representation of physical operations in real time. In underground mining, where conditions are extremely variable and risky, digital twins offer significant opportunities to improve safety, efficiency, and decision-making. However, their adoption still faces technical and operational challenges. This text explores the future directions of this technology, analyzing its potential and key areas of development.

### 8.1. Advanced Integration with IoT and Smart Sensors

One of the main focuses of development is the deeper integration of digital twins with IoT sensor networks and smart devices. In underground mining, this involves the following:

- Real-time monitoring of environmental conditions (gases, temperature, humidity).
- Precise tracking of equipment and personnel using wearable sensors.
- Predicting machine failures through vibration and wear analysis.

In the future, digital twins are expected to process this data more autonomously, using AI to generate preemptive alerts [71].

### 8.2. Simulation and Optimization of Operations

Digital twins will enable more advanced simulations to optimize underground mining processes, including the following:

- Dynamic tunnel and mining planning, adjusting strategies based on updated geological data.
- Virtual testing of new technologies, such as autonomous equipment or ventilation systems, before their physical implementation.
- Modeling risk scenarios, such as landslides or fires, to improve emergency protocols.

These capabilities will reduce costs and increase productivity, while minimizing occupational hazards [72].

### 8.3. Artificial Intelligence and Machine Learning

The future of digital twins in underground mining is closely tied to the advancement of AI and ML. Some promising applications include the following:

- Predictive diagnosis of failures in critical equipment, such as drilling rigs or transportation systems.
- Automated recommendations to improve energy efficiency or mineral logistics.
- Analysis of historical data to identify patterns and optimize long-term processes.

As algorithms become more precise, digital twins will be able to make semi-autonomous decisions in real time [71].

### 8.4. Augmented Reality and Immersive Visualization

Another important direction is the incorporation of AR and VR technologies to enhance interaction with digital twins. This could include the following [46]:

- Three-dimensional visualization of underground mines in control centers, allowing for more intuitive monitoring.
- Virtual training for workers, simulating real-life conditions without exposing them to hazards.
- Remote assistance from experts, who could virtually “enter” the mine to resolve technical issues.

### 8.5. Interoperability and Global Standards

For digital twins to reach their full potential, establishing interoperability standards across different systems and platforms will be crucial. This involves the following [73]:

- Compatibility between software from various vendors, avoiding information silos.
- Cybersecurity protocols, given the increasing digitalization of mining operations.
- Regulations and standards that guide the ethical and efficient implementation of this technology.

### 8.6. Sustainability and Improved Safety

Environmental and cybersecurity are key to the proper use and implementation of digital twins for underground mining, and in this regard, at least the following key aspects should be considered in these areas [5,20]:

- Environmental footprint: simulation of strategies to reduce emissions and energy consumption.
- Proactive safety: risk detection through pattern analysis (e.g., gas buildup, pillar instability).

### 8.7. Pending Challenges

There are pending issues in the application of digital twins, such as (i) data quality, as errors in sensors or delays can affect the reliability of the twin, (ii) cybersecurity, or the protection of critical systems against attacks, and (iii) cost and complexity, such as implementation in small or traditional mining companies [74].

Implementing a digital twin in an underground mine first faces formidable hardware barriers, which constitute the sensory backbone of the system. The underground environment is inherently hostile, characterized by humidity, abrasive dust, constant vibrations, and the risk of explosions, requiring all IoT sensors and devices to be not only highly accurate but also robust, intrinsically safe, and often impact-resistant. This significantly increases their cost and complexity. Furthermore, connectivity is a monumental challenge, as rock blocks conventional radio signals such as Wi-Fi or Bluetooth, necessitating the deployment of specialized infrastructure such as “Leaky Feeder” cable systems or mining-specific LTE/5G networks, which entail costly investments in cables and repeaters. Furthermore, reality capture using LIDAR scanners or drones is hampered by the lack of a GPS, requiring inertial navigation systems, and the need for frequent model updates generates a massive volume of data. Finally, this entire infrastructure relies on a high-capacity network and formidable computing power, often requiring local data centers (edge computing) to process information in real time, all backed up by reliable uninterruptible power systems in an environment where power outages can blind the twin in a matter of seconds.

Once the physical challenges are overcome, software hurdles emerge as the “brain” that must make sense of the data, presenting equally complex challenges. Integration and interoperability are perhaps the biggest stumbling block. A mine operates with a heterogeneous mix of machinery and software from multiple vendors, each with its own protocols and standards. Getting all these systems to “speak the same language” and ingest data harmonized into a unified platform requires the use of cutting-edge standards and considerable engineering effort, with the added risk of being locked into a closed ecosystem of a single vendor. Furthermore, the very heart of the twin—modeling and simulation—collide with the extreme geotechnical and geomechanical complexity of the rock mass. Accurately simulating its behavior in real or near-real time requires advanced algorithms and massive computational power, where any error or excessive latency in the model can lead to erroneous recommendations with potentially catastrophic consequences for safety and operations. Managing the massive volume of data (Big Data) efficiently and securing the platform against cyberattacks, which could paralyze the entire operation, requires a software landscape that must be simultaneously powerful, integrative, and extremely secure.

However, even with the most advanced technology, the human factor is often the most underestimated and critical obstacle to success. Implementing a digital twin frequently encounters strong resistance to cultural change. Workers and engineers with decades of practical experience may perceive this technology as a threat to their professional judgment and their jobs, which generates distrust of automated recommendations and can lead to ignoring system alerts. At the same time, there is a profound skills gap. The mine needs new profiles that did not previously exist, such as data scientists, simulation engineers, and IoT specialists, who are scarce in the market and expensive to hire. At the same time, it is imperative to cross-train existing personnel—from geologists to operators—so they can interpret the twin’s visualizations and data and integrate them into their daily decision-making. This is not a simple course but a fundamental shift in mindset. Finally, without a clear redefinition of operational processes and strong leadership to communicate strategic vision and act as a project champion, the digital twin risks becoming an underutilized tool, a “pretty dashboard” disconnected from real decision-making, thus failing to fulfill its transformative purpose.

## 9. Conclusions

The insertion of digital twins in underground mining marks a historic turning point, transitioning from a reactive and fragmented operational model to a proactive, integrated, and inherently intelligent one. This article has demonstrated that this technology goes far beyond simple 3D visualization; it serves as the core of a connected, self-aware mine that can optimize its operations autonomously.

The implementation of digital twins in underground mining represents a transformative advance for the industry, enabling more efficient, safe, and sustainable management of operations. By integrating real-time data with dynamic virtual models, this technology facilitates process optimization, failure prediction, and decision-making based on predictive analytics. It also contributes to reducing occupational hazards and improving resource utilization, aligning with Mining 4.0 standards.

As previously presented, 10 experts with over 8 years of experience in underground mine design, construction, and operation evaluated the advanced multi-layer digital twin conceptual framework for underground mining, providing positive feedback on its potential, as shown in the evaluation results.

The digital twin consolidates information from multiple sources (geological, geotechnical, operational, environmental, and maintenance) into a single, unified platform. This eliminates data silos and provides engineers and managers with a holistic, real-time view of the entire operation, enabling faster, more accurate, and data-driven decision-making.

The ability to simulate scenarios, predict structural failures, model rock behavior, and plan evacuations in a virtual environment constitutes the most powerful safety tool developed in decades. Digital twin technology transforms safety from a reactive concept (responding to incidents) into a truly predictive and proactive approach, thus protecting the most valuable asset: people.

The mining industry of the future is autonomous, and the digital twin is the cornerstone that makes it possible. By acting as the “brain” of the operation, it can orchestrate fleets of autonomous equipment, intelligent ventilation systems, and automated processes, creating an ecosystem that continuously self-regulates and optimizes itself.

However, the adoption of digital twins in underground mining requires overcoming challenges such as data standardization, personnel training, and investment in technological infrastructure. As these tools are refined with advances in artificial intelligence, IoT, and cloud computing, digital twins will become a strategic pillar for the mining industry of the future, boosting productivity and sustainability in complex underground environments.

Ultimately, digital twins not only modernize the mining industry but also open new possibilities for smarter and more responsible exploitation of mineral resources. The future of digital twins in underground mining points toward more autonomous, interconnected, and predictive systems, with a strong focus on safety and sustainability. However, their success will depend on advances in IoT, AI, interoperability, and companies’ ability to adopt these technologies in a scalable manner.

In conclusion, digital twin technology is not just another technology; it is the definitive operating system for 21st-century underground mining. Its implementation is no longer a competitive advantage but rather a strategic necessity for companies seeking to operate more safely, efficiently, sustainably, and resiliently in an increasingly complex geological environment. Those who adopt and master this technology will be leading the next mining revolution.

## Figures and Tables

**Table 1:** Searching the index of the literature search method according to the mentioned databases.

Searching Index	Content
Time	2020 (March)–2025 (July)
Database	Scopus and Web of Science
Title/Subject-Abstract	“Digital Twins” AND “Underground Mine” AND “Mining”
Publication Type	“Article” and “Review Article”
Data Extraction Index 1	Technological advances
Data Extraction Index 2	Integration with Industry 4.0 Concept

**Table 2:** Literature review.

#	Authors Reference	Technological Advances	Sensors	AI	ML	IoT	Big Data	CC	AU	RO
1	[21]	This paper studies the development of a customized digital twin framework for the mining industry. Also, this research provides model selection tools to aid digital twin construction.	X	X	X					
2	[22]	This research found that digital twin technology can be applied across all mining project phases and can provide added value to improve multiple ESG factors and measure them.		X						
3	[23]	This study provides valuable insights into fully integrated digital twin mining systems, which will significantly improve mining efficiency and sustainability.	X	X		X				
4	[24]	This paper intends to shed light on the state of art of DT case studies focusing on concept, design, and development.		X		X	X	X		
5	[25]	This article discusses the current state of machine learning and Big Data in digital twins and finds that advancement in these technologies has impacted the adoption and ideal implementation of digital twins.			X		X			
6	[26]	This paper studies how digital twins can be distinguished from other types of digital modeling and simulation.	X			X				
7	[27]	Through a systematic literature review and a thematic analysis of 92 digital twin publications from the last ten years, this paper provides a characterization of the digital twin, identification of gaps in knowledge, and required areas of future research.							X	X
8	[28]	This paper summarizes the development trends and directions of digital twin research.	X			X				
9	[29]	At this article challenges to digital twin implementation are identified and categorized.		X	X					
10	[30]	This paper studies how technologies such as IoT, cloud computing, and blockchain have increased the potential of digital twins and how digital twins should include the “things” and “humans” from the entire supply chain.				X		X		
11	[31]	This paper catalogs supporting tools aligned with the technical specifications stipulated in DTs’ functional capabilities, aiding developers in devising implementation strategies.		X	X					
12	[32]	This article outlines an IoT and digital twin prototype for automating large-scale conventional mines, referencing architecture for mining to model, simulate, and control the underground physical world virtually.				X				X
13	[33]	In this paper, we focus on the construction of DTs. More specifically, we focus on determining (methodologically) how to design, create, and connect physical objects with their virtual counterpart. We explore the problem into several phases: from functional requirement selection and architecture planning to integration and verification of the final (digital) models.	X	X		X				
14	[34]	This study fills this gap by systematically reviewing DT research, focusing on three interrelated aspects: DT applications, architectural layers, and security.		X						
15	[35]	This paper studies the incorporation of sensing and machine learning into reflective twins for dynamic updates, describing progress and insights for multi-criteria decision-making for intervention.	X		X					

**Table 3:** Characterization of experts who participated in the research.

No.	Occupation	Profession	Labor Camp	Country of Residence	Experience (Years)
1	Superintendent of Underground Mine	Mining Engineer, MSc	Underground mining operation	Peru	20
2	Underground Mining Engineer	Mining Engineer	Underground mining operation	Chile	8
3	Underground Mining Engineer	Mining Engineer	Underground mining operation	Chile	10
4	Underground Mining Consultant	Mining Engineer, PhD	Design, construction and operation of underground mines	Peru	25
5	Underground Mining Engineer	Mining Engineer, MBA	Underground mining operation	Peru	12
6	Underground Mining Manager	Mining Engineer, MSc	Design, construction and operation of underground mines	Chile	25
7	Underground Mining Engineer	Mining Engineer, MBA	Safety in underground mines	Chile	14
8	Superintendent of Underground Mining	Mining Engineer, MSc	Design, construction and operation of underground mines	Chile	18
9	Underground Mining Engineer	Mining Engineer, MBA	Safety in underground mines	Chile	10
10	Underground Mining Engineer	Mining Engineer	Underground mining operation	Peru	12

**Table 4:** Quality statements.

No.	Quality Dimension	Statement
1	Technology	The technological dimension of a digital twin refers to the set of tools, platforms, standards, and software and hardware architectures that enable the creation, maintenance, operation, analysis, and ongoing management of the virtual representation of a physical object or system.
2	Operation	The operational dimension refers to the practical and applied use of the digital twin to monitor, control, optimize, and automate the operations of a physical system in real time. It is the dimension that connects the data and the virtual model with decision-making and concrete actions in the real world.
3	Productivity	The productivity dimension refers to the ability of the digital twin to generate measurable improvements in the efficiency, effectiveness, and performance of a process, system, or organization. It involves quantifying how the digital twin enables organizations to achieve more with less: increasing production, improving quality, reducing costs, and optimizing the use of resources (time, materials, energy, and human capital).
4	Safety	The security dimension refers to the set of protocols, technologies, and practices implemented to protect the safety of mine workers. Its objective is to prevent physical, cyber, and operational risks, ensuring that decisions made based on the digital twin do not compromise the safety of people, equipment, or the environment.
5	Sustainability	The sustainability dimension refers to the ability of the digital twin to monitor, simulate, optimize, and report on the environmental and social performance of a physical asset or process throughout its entire lifecycle. Its objective is to minimize negative environmental impact (emissions, waste, resource consumption) and maximize positive social impact, all while remaining economically viable.
6	Economy	The economic dimension refers to the analysis of the costs, benefits, return on investment (ROI), and business models associated with the implementation and use of a digital twin. It evaluates how the digital twin contributes to an organization’s financial viability, profitability, and competitive advantage, whether through cost reduction, asset optimization, or the creation of new revenue streams.

**Table 5:** Evaluation statements with the expert panel.

ID	Quality Dimension	Ask
Q1	Technology	The digital twin in an underground mining context is differentiated from a traditional 3D model.
Q2	Technology	Technologies (IoT, AI, sensors, etc.) are essential to developing a functional digital twin in an underground mine.
Q3	Operation	There may be greater technical and logistical challenges when implementing a digital twin in an underground mining operation.
Q4	Operation	Real-time data from machinery, geology, and personnel, if integrated into a digital twin
Q5	Productivity	Infrastructure requirements (connectivity, hardware, software) may be necessary to maintain an efficient digital twin.
Q6	Productivity	A digital twin could optimize production planning and logistics in underground mining.
Q7	Safety	Decision-making in a digital twin is improved in critical situations, such as structural failures or unexpected geological changes.
Q8	Safety	The digital twin does predict events such as landslides or floods through simulations.
Q9	Safety	The digital twin contributes to worker safety in underground environments
Q10	Sustainability	A digital twin plays an important role in monitoring environmental conditions (air quality, vibrations, temperature).
Q11	Sustainability	A digital twin could offer sustainability benefits (waste reduction, energy efficiency)
Q12	Economy	A digital twin could generate ROI (Return on Investment) in an underground mine.
Q13	Technology	Technological advances (e.g., 5G, edge computing) could power digital twins in the coming years
Q14	Operation	A digital twin considers the training of staff to operate and take advantage of these technological tools.
Q15	Operation	The digital twin could serve as the foundational basis for achieving fully autonomous underground mining operations in the future.

**Table 6:** Evaluation of hypotheses H1 to H14 with Wilcoxon statistics.

Ask	N for the Test	Wilcoxon Statistic	*p*-Value
Q1	7	60.0	0.001
Q2	10	103.0	0.001
Q3	8	75.0	0.001
Q4	9	88.0	0.001
Q5	9	88.0	0.001
Q6	10	103.0	0.001
Q7	10	103.0	0.001
Q8	6	52.0	0.002
Q9	10	103.0	0.001
Q10	8	75.0	0.001
Q11	7	60.0	0.001
Q12	9	88.0	0.001
Q13	10	103.0	0.001
Q14	10	103.0	0.001

N: Number of experts considered for the test.

**Table 7:** Spearman correlation matrix.

	Q1	Q2	Q3	Q4	Q5	Q6	Q7	Q8	Q9	Q10	Q11	Q12	Q13
**Q2**	0.23												
**Q3**	−0.53	0.20											
**Q4**	0.31	0.26	0.36										
**Q5**	−0.21	−0.12	0.16	−0.31									
**Q6**	0.44	0.09	0.06	0.25	0.44								
**Q7**	0.33	−0.18	0.66 *	0.44	0.37	0.22							
**Q8**	0.08	0.05	0.12	−0.27	0.62 *	0.25	0.59 *						
**Q9**	0.21	−0.30	0.36	0.79 *	0.09	−0.08	0.04	0.38					
**Q10**	0.61	0.23	−0.19	0.44	−0.22	0.56 *	0.26	0.10	0.08				
**Q11**	0.14	−0.15	0.69 *	−0.19	0.06	0.33	0.12	0.33	0.11	0.34			
**Q12**	0.42	0.81 *	0.12	0.08	−0.07	−0.15	−0.20	0.80 *	0.36	0.22	−0.05		
**Q13**	0.56	0.27	−0.38	0.71 *	−0.22	0.22	0.44	−0.13	0.11	0.72 *	0.21	0.16	
**Q14**	0.09	0.19	−0.33	0.07	0.14	0.08	0.35	−0.30	−0.11	0.29	0.33	0.68 *	−0.05
***p*-value < 0.05**													

## Abbreviations

The following abbreviations are used in this manuscript.

DT	Digital Twin
AI	Artificial Intelligence
ML	Machine Learning
CC	Cloud Computing
IoT	Internet of Things
AU	Automation
RO	Robotics
DSS	Decision Support System
ROI	Return On Investment
AR	Augmented Reality
VR	Virtual Reality
LiDAR	Light Detection and Ranging
CAD	Computer-Aided Design
FEM	Finite Element Model
KPI	Key Performance Index
PLCs	Programmable Logic Controllers
LHD	Load Haul Dump
BIM	Building Information Modeling
SCADA	Supervisory Control and Data Acquisition
